# Proteomic Analysis of Skeletal Muscle in Insulin-Resistant Mice: Response to 6-Week Aerobic Exercise

**DOI:** 10.1371/journal.pone.0053887

**Published:** 2013-01-09

**Authors:** Hairui Yuan, Yanmei Niu, Xiaolei Liu, Fengying Yang, Wenyan Niu, Li Fu

**Affiliations:** 1 Department of Rehabilitation and Sports Medicine, Tianjin Medical University, Tianjin, China; 2 Department of Immunology, School of Basic Medical Science, Tianjin Medical University, Tianjin, China; 3 Department of Physiology, School of Basic Medical Science, Tianjin Medical University, Tianjin, China; Universidad Europea de Madrid, Spain

## Abstract

Aerobic exercise has beneficial effects on both weight control and skeletal muscle insulin sensitivity through a number of specific signaling proteins. To investigate the targets by which exercise exerts its effects on insulin resistance, an approach of proteomic screen was applied to detect the potential different protein expressions from skeletal muscle of insulin-resistant mice after prolonged aerobic exercise training and their sedentary controls. Eighteen C57BL/6 mice were divided into two groups: 6 mice were fed normal chow (NC) and 12 mice were fed high-fat diet (HFD) for 10 weeks to produce an IR model. The model group was then subdivided into HFD sedentary control (HC, n = 6) and HFD exercise groups (HE, n = 6). Mice in HE group underwent 6 weeks of treadmill running. After 6 weeks, mice were sacrificed and skeletal muscle was dissected. Total protein (n = 6, each group) was extracted and followed by citrate synthase, 2D proteome profile analysis and immunoblot. Fifteen protein spots were altered between the NC and HC groups and 23 protein spots were changed between the HC and HE groups significantly. The results provided an array of changes in protein abundance in exercise-trained skeletal muscle and also provided the basis for a new hypothesis regarding the mechanism of exercise ameliorating insulin resistance.

## Introduction

Insulin resistance (IR) is critical to the pathogenesis of the metabolic syndrome, which precedes the development of type 2 diabetes mellitus (T2DM) and cardiovascular disease [1], [2]. As the predominant tissue for insulin-stimulated glucose and lipid disposal, skeletal muscle is crucial for the development of whole-body IR [3]. Numerous studies over the past few decades have revealed an array of abnormalities in insulin action in the skeletal muscle of obese and patients with T2DM. In a state of insulin resistance, insulin-stimulated glucose disposal in skeletal muscle is markedly impaired, which may be associated with impaired insulin signaling, multiple post-receptor intracellular defects, and reduced glucose oxidation and glycogen synthesis [4]. Although the exact mechanism of IR in skeletal muscle has not been fully elucidated, it seems clear lack of physical exercise and constant over-nutrition, appear to have triggered the escalating incidence of IR. Recent studies have reported that an increased intramyocellular fat content and lipid-derived mediators including diacylglycerol (DAG) or ceramide may play a key role in the development IR [5], [6]. The accumulation of fat inside the skeletal muscle could originate from an excess supply of free fatty acid (FFA) to the muscle and a decreased rate of fatty acid oxidation in skeletal muscle cells that is either induced by impaired mitochondrial function or by lower demand for ATP reducing electron flux through mitochondria; or some combination of the two. In addition, a large number of studies reveal that low-grade inflammation and oxidative stress were directly involved in the pathogenesis of IR [7], [8].

Regular physical activity has been viewed as one of the best non-pharmacological strategies for the prevention or attenuation of IR and T2DM. Exercise activates a number of transcriptional regulators and serine-threonine kinases in skeletal muscle that contribute to metabolic reprogramming, including improved mitochondrial function, increased fatty acid oxidation, and enhanced insulin action on the skeletal muscle [9]–[11], but the exact targets by which exercise training stimulates the favorable phenotype are not fully understood.

Using microarray analysis, we have discovered a global gene expression profile in skeletal muscle of IR mice that underwent aerobic exercise [12]. However, the mRNA levels for a gene may not be in line with the abundance of the proteins encoded by that gene, which are a closer reflection of the level of activity of a biological pathway [13]; therefore, it is necessary to detect the global protein expression for the further study of IR. The advance of 2-D gel, mass spectrometry, and bioinformatics have made proteomic methods valuable tools for quantifying differences in protein abundance between different physical conditions or treatments. Although several reports have described the skeletal muscle proteome and identified a number of abnormalities in obesity and T2DM [14], [15], the application of proteomics to investigate the response of skeletal muscle to exercise training is in its infancy. Holloway et al detected 256 gel spots of which 20 proteins were differential expressed after 6-week interval training [16]. Using the 2-D DIGE analysis, Egan et al investigated the mitochondrial proteome from human skeletal muscle in response to 14 consecutive days of endurance exercise training, revealing changes in phosphotransfer system, mitochondrial protein synthesis machinery, tricarboxylic acid cycle proteins and metabolic enzymes [17]. However, the global protein expression profile of the skeletal muscle response of IR mice to aerobic exercise has not yet been reported. The purpose of this study is to develop a global profile of protein abundance changes in IR mice that underwent a 6-week aerobic exercise program to provide data that can serve as a basis for generating novel hypotheses regarding the molecular mechanisms that alleviate IR by aerobic exercise.

## Materials and Methods

### Animals

All animal protocols were approved by the Tianjin Medical University Animal Care and Use Committee under the guidelines of the Chinese Academy of Sciences. A total of eighteen male, 4-week old C57BL/6 mice were purchased from the Institute of Chinese Military Academy of Medical Science at 12.34±1.28 g in mass. Upon arrival, the mice were housed in a controlled environment with a reversed 12/12 h light-dark cycle and free access to food and water. After 1 week of acclimation, the mice were randomly divided into an NC group (*n* = 6) and an HFD group (*n* = 12), fed an NC and an HFD (45% calories from fat, #D12451, Research Diets), respectively, for up to 10 weeks. Thereafter, the HFD group randomized into HFD control (HC, *n* = 6) and HFD exercise group (HE, *n* = 6), and these two groups continually fed an HFD continually for up to 16 weeks. Their body weight was measured once a week.

### Exercise Protocol

Mice randomized to the HE group underwent several acclimation exercise sessions on a motorized treadmill (electrical stimulus) at 10 m/min (0% grade) for 20 min during the first week. Thereafter, the mice underwent 6 weeks of treadmill training at 12 m/min (75% VO_2_ max) for 60 min/day, 5 days/week on a 0% grade [18]. To eliminate any acute effect of the last exercise bout, the experimental procedures were carried out 48 hours after the last training session.

### Oral Glucose Tolerance Test (OGTT), Fasting Serum Insulin (FIN), and Metabolic Parameters

Mice were fasted overnight prior to administration of OGTT. D-glucose (Sigma, USA) was dissolved in ddH_2_O and administered orally to the fasted mice (2 g/kg of body weight) using a 20-gauge stainless steel gavage feeding needle. Samples of whole-blood (2–3 µl each) were collected from a tail clip bleed immediately before and 15, 30, 60 and, 120 min after administration of glucose. Blood glucose levels were measured using the Blood Glucose Monitoring System (One Touch, LifeScan, USA). For FIN, the blood samples (200–300 µl) were taken from vena caudalis, Insulin level was measured by insulin ELISA kit (Millipore). High-density lipoprotein (HDL), total cholesterol (TC) triglycerides (TG) and free fatty acid (FFA) were determined by enzymatic kits (Nanjing Jiancheng Bioengineering Institute, China).

### Sample Preparation

Mice were fasted for 14–16 hours, after which they were sacrificed humanely by injecting with 40 mg of anesthetic (hydral) per 100 g of body weight; quadriceps femoris was then removed and frozen with liquid nitrogen; a blood sample was collected; and serum was separated via centrifuge and stored at −80°C. Six mice were selected randomly for 2D gel analysis, citrate synthase activity and western blot in the present study. For 2-D gel analysis, quadriceps femoris (*n = *6, each group) was pulverized in liquid nitrogen and then homogenized on ice in 5 volumes lysis buffer, containing: 40 mM Tris-HCl, 7 M urea, 2 M thiourea, 4% CHAPS, 1% DTT, 1 mM EDTA, and protease inhibitor cocktail (Sigma, USA). After centrifugation at 14000 g, 4°C for 20 min, the supernatant was decanted and stored at -80°C. Protein concentration was measured by using Bradford assay.

### 2-DE

Supernatant, containing 100 µg proteins, was separated by 2-D gel. The first dimensional IEF was performed with the IPGphor IEF system (GE Healthcare, Life Sciences, USA), as previously described [19]. Briefly, Immobiline™ pH 3–10 linear DryStrips were rehydrated for 10 h using reswelling buffer (8 M urea, 2% CHAPS, 0.02 M DTT) and 0.5% IPG Buffer. The voltage during IEF was applied according to the following procedure: 500 V for 1 h, 1000 V for 1 h, and 8000 V for 10 h. After IEF, the strips were equilibrated for 15 min in equilibration solution I (1.5 M Tris-HCl, pH 8.8, 30% glycerol, 6 M urea, 2% SDS, bromophenol blue trace, 20 mM DTT). The strip was then transferred to equilibration solution II (1.5 M Tris-HCl pH 8.8, 30% glycerol, 6 M urea, 2% SDS, bromophenol blue trace, 100 mM iodoacetamide) for 15 min. The second dimensional SDS–PAGE was performed using 13% polyacrylamide gel without a stacking gel in the PROTEAN II cell (Bio-Rad Laboratories, USA). Electrophoresis was stopped when the bromophenol blue dye front reached the bottom of the gel. One 2-D gel was performed each sample, 6 samples per group.

### Staining

Gels were stained with silver for the analytical gels used for spot quantitation. For preparative gels, a glutaraldehyde-free method designed to optimize subsequent spot excision and protein extraction for LC-MS/MS was used as follows: Gels were fixed in 40% alcohol and 10% acetic acid for 30 min. They were then washed 3 times in 35% alcohol for 20 min each, followed by sensitization in 0.02% Na_2_S_2_O_3_ for 30 min. Gels were then washed 3 times in distilled H_2_O for 5 min each and stained in 0.25% silver nitrate and 0.04% formaldehyde solution for 20 min. Gels were washed twice in distilled H_2_O for 1 min each and developed in staining solution (2.5% Na_2_CO_3_, 0.04% formaldehyde solution). Staining was stopped by incubating the gels in 1.46% EDTA. The preparative gels for subsequent spot excision and protein extraction for MALDI-TOF-MS were stained with 0.1% CBB G-250 in 50% methanol and 10% acetic acid.

### Image Analysis

Silver-stained gels were scanned using an image scanner (GE Healthcare, Life Sciences, USA) and ImageMaster™ 2-D Platinum (GE Healthcare, Life Sciences, USA) was used for the analysis of the resulting protein pattern. Filtered images were created, followed by automatic spot detection; spots that the software indicated as low quality were manually redrawn using a freeform tool to outline the spot. A reference gel was selected and a “match set” was constructed by matching spots of each gel to the reference gel. The quantity of each spot was normalized by total valid spot intensity. Protein spots were selected as being significantly different (*p*<0.05) if the expression was altered by 1.5 fold compared with the control sample.

### In-gel Digestion

Spots were excised from the gel stained with silver or stained with CBB. Spots was transferred into a centrifuge tube and washed in 200 µl aliquots of 20 mM NH_4_HCO_3_ in 50% acetonitrile for 30 min to be destained (or 50–100 µl aliquots of 30 mM potassium ferricyanide in 50% 100 mM sodium hyposulfite for silver stained gel). The gel pieces were then vacuum dried and rehydrated at 4°C for 30 min in 10 µl digestion solution (25 mM NH_4_HCO_3_ and 0.01 µg/ µl modified sequence-grade trypsin); 4–10 µl of digestion solution without trypsin was then added to keep the gel pieces wet during the digestion. After overnight incubation at 37°C, the digestion was stopped with 5% TFA for 20 min. Peptides were extracted by 5% TFA at 45°C for 60 min and then by 2.5% TFA/50% acetonitrile at 45°C. The combined supernatants were evaporated in the SpeedVac Vacuum for mass spectrometric analysis.

### Protein Identification by MALDI-TOF-MS

For MALDI-TOF-MS experiments, spots from the CBB-stained gels were obtained on an UltraFlex (Bruker-Franzen Analytik, Bremen, Germany) in positive ion mode at an accelerating voltage of 20 kV with the matrix of alpha-Cyano-4-hydroxycinnamic acid. Peptide mass fingerprinting obtained was used to search through the SWISS-PROT and NCBInr databases by the Mascot (www.matrixscience.com) Server. The enzyme specificity was set as trypsin allowing 1 missed cleavage, carbamidomethyl modification of cysteine (variable), oxidation of methionine (variable) with an m/z tolerance of ±0.1 Da.

### Protein Identification by LC-MS/MS

For the spots from silver stained gels, LC-MS/MS experiments were performed on a Synapt High Definition Mass Spectrometry (Waters Corp., Milford, USA) equipped with a nanoACQUITY UPLC system (Waters Corp., Milford, USA) as previously described [20]. In brief, a Symmetry C18 precolumn (5 µm, 180 µm×20 mm) and an ethylene bridged hybrid (BEH) C18 analytical reversed-phase column (1.7 µm, 75 µm × 250 mm) were used for the reversed-phase UPLC analysis. The samples were initially transferred with an aqueous 0.1% formic acid solution to the precolumn. Mobile phase A consisted of 0.1% formic acid in water, and mobile phase B was composed of 0.1% formic acid in acetonitrile. The peptides were separated with a linear gradient of 3–40% mobile phase B over 65 min at 200 nl/min followed by 10 min at 85% mobile phase B. Analysis of tryptic peptides was performed using a SYNAPT high definition mass spectrometer. The mass range was from MS: 350 to 1400, MS/MS: 50 to 2000. The amino-acid sequences of the peptides were deduced with the peptide sequencing program MasSeq. The database search was finished with the MS/MS Ion Search in the Mascot Search engine (www.matrixscience.com). The search parameters were as follows: Variable modifications including: carbamidomethyl (C), oxidation (M); allowing three missed cleavage; peptide mass tolerance ±0.5 Da; fragment ion tolerance ±0.5 Da.

### Citrate Synthase Activity

Quadriceps femoris samples (n = 6, each group) were placed in ice-cold lysis buffer (50 mM Tris, pH 7.4, 0.15 M KCl), homogenized and centrifuged at 13000 g for 10 min. The supernatants were taken, and the protein concentration was measured using Bradford assay. The reaction catalyzed by citrate synthase as follows: Acetyl-CoA+oxalacetate+H_2_O → citrate+CoA-SH (colorimetric reaction: CoA-SH+DTNB → TNB+CoA-S-S-TNB). Citrate synthase activity was determined by measuring the appearance of the yellow product (TNB), which is observed spectrophotometrically by measuring absorbance at 412 nm. The citrate synthase reagent consisted of 0.1 mM DTNB, 10% Triton X-100, 0.31 mM acetyl CoA (Sigma, USA), and muscle sample (5 µg). The reaction regent (200 µl) included also 10 µl 10 mM oxalacetate (Sangon Biotech, China) that was added to start the reaction. The absorbance changes were measured every 20 s over 3 min at 412 nm for determine of the citrate synthase activity (Biotek, Synergy HT Multi-Mode Microplate Reader, USA). All assays were carried out at 30°C. Citrate synthase from porcine heart (C-3260, Sigma, USA) was used as a standard for assay calibration.

### Western Blot Analysis

Quadriceps femoris lysates of 6 mice in each group were prepared with NP-40 buffer, homogenized, and centrifuged at 12000 g for 20 min. The extract was diluted in SDS loading buffer and heated at 70°C for 10 min. The sample (50 µg) was subjected to electrophoresis in 12% SDS–PAGE at 110 V. The gel was transferred onto 0.45 µm PVDF membrane at 300 mA for 2 h. The membrane was then blocked with 10% non-fat milk in TBST (10 mM Tris-HCl, 150 mM NaCl, pH 7.5, 0.1% Tween 20) for 1 h at room temperature. After washing with TBST, the membrane was probed with primary antibody overnight at 4°C. The following antibodies were used in this study: Rabbit anti-mouse Hsp25, Fabp4 (Cell Signaling Technology), MHC IIb (Proteintech, USA), Trim72 (Everest Biotech, UK), Skeletal muscle actin and β-tubulin (Abcam). After washing with TBST, the membranes were incubated with HRP-conjugated anti-rabbit secondary antibody (Cell Signaling Technology) for 1 h at room temperature and developed using an ECL kit according to the manufacturer’s instructions (GE Healthcare, Life Sciences, USA).Western blot was analyzed by scanning with a Scanner and digitalizing using image analysis software (Quantity One).

### Statistical Analysis

The results of spot volume intensities on 2-D gels (n = 6, each group) were compared by one-way ANOVA using ImageMaster™ 2-D Platinum. Body weight, fasting insulin levels, plasma parameters and skeletal muscle protein levels were analyzed with one-way ANOVA using the Statistical Package for the Social Sciences (SPSS) program. The data were expressed as the mean ± SEM. Group means were considered to be significantly different at *p*<0.05.

## Results

### Body Weight, Fasting Serum Insulin (FIN), OGTT, Metabolic Profiles and Citrate Synthase Activity

**Figure 1:**
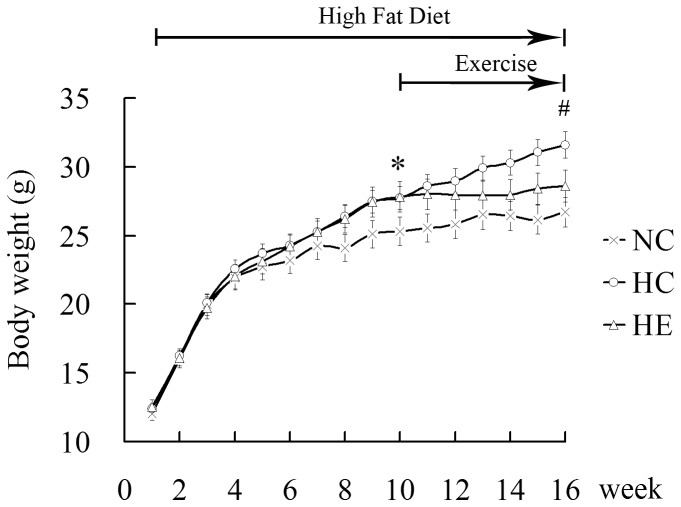
Body weight in the experimental procedure. Values are means ± SEM (n = 6, each group). *: HC (HFD control) vs. NC (normal chow control) p<0.05; ^#^: HE (HFD exercise) vs. HC p<0.05.

Mice were randomly divided into two groups, with 6 mice fed an NC and 12 mice fed an HFD. The latter was subdivided into HC (*n* = 6) and HE (*n* = 6). Changes in body weight between groups during the experimental period are shown in Fig. 1. The body weights of mice were the same at the beginning of this study; however, they began to diverge after 4 weeks. There was significant difference between NC and HC up to 10 weeks, such that HC mice were heavier (p<0.05) than NC at the 10^th^ week. As expected, the body weight of mice in the HE group that underwent a 6-week aerobic exercise regimen decreased significantly compared with HC. At the 16^th^ week, FIN of mice in HC was increased significantly compared with NC; however, it was decreased significantly after 6 weeks of aerobic exercise in HE compared with HC (Table 1). Biochemical parameters in the plasma of mice are also shown in Table 1. Six-week aerobic exercise improved plasma lipid profiles, including reduced FFA, TC, and TG levels, and increased HDL levels.

**Table 1:** Insulin level and plasma lipid parameters after 6-week aerobic exercise.

	NC	HC	HE
Insulin (ng/mL)	0.356±0.072	0.556±0.081*	0.402±0.062#
FFA (mmol/L)	1.124±0.074	1.517±0.136*	1.143±0.139#
HDL (mmol/L)	2.310±0.197	2.452±0.175	3.679±0.203#
TC (mmol/L)	2.293±0.196	3.586±0.328*	2.994±0.329#
TG (mmol/L)	0.579±0.081	0.946±0.145*	0.639±0.129#

Values are means ± SEM (n = 6, per group).

* P<0.05 HFD control (HC) vs. normal chow (NC).

# P<0.05 HFD exercise (HE) vs. HC.

FFA: Free fatty acid; HDL: High-density lipoprotein; TC: Total cholesterol; TG: Triglycerides.

**Figure 2:**
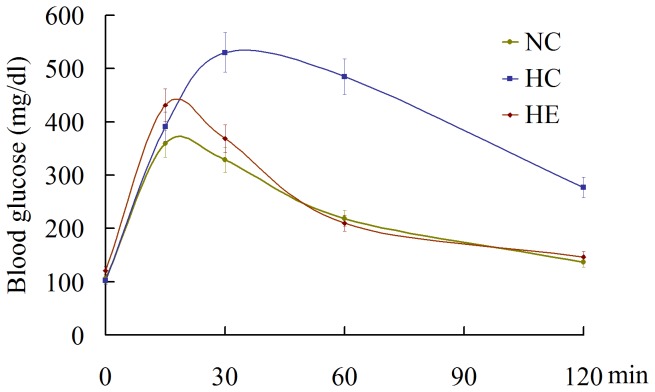
Evaluation of glucose tolerance by OGTT. Oral glucose tolerance test was performed in each group (n = 6) of mice after 6 weeks of aerobic exercise. Values are means ± SEM. NC = normal chow control; HC = HFD control; HE = HFD exercise.

To evaluate glucose homeostasis and tolerance, we used a 2-hour OGTT, shown in Fig. 2. Blood glucose levels in the NC group peaked at ∼370 mg/dl and approached baseline level by 120 min after glucose challenge, whereas the HC group peaked at ∼530 mg/dl and remained elevated compared with the NC group. The glucose level of peak in HE was decreased significantly and the glucose level at 120 min approached baseline level.

**Figure 3:**
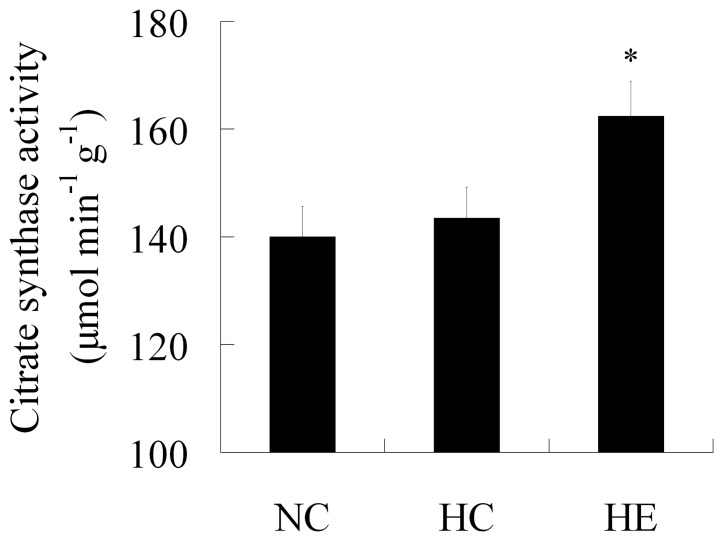
Citrate synthase activity in each group. Citrate synthase activity levels in the quadriceps muscle of mice from NC, HC and HE, respectively. Values are shown as means ± SEM (n = 6, each group). *:P<0.05 vs. HC.

To determine whether skeletal muscle metabolic capacity was enhanced by 6-week treadmill training, the citrate synthase activity was measured. Data of citrate synthase activity is present in Fig. 3. There is no significantly difference between NC (140.08±8.50 µmol/min/g) and HC group (143.52±7.92 µmol/min/g). As expected, citrate synthase activity was significantly elevated in HE group (162.42±8.49 µmol/min/g, p<0.05) compared with HC group.

### Overview of Proteomic Analysis of Skeletal Muscles of All Groups

**Figure 4:**
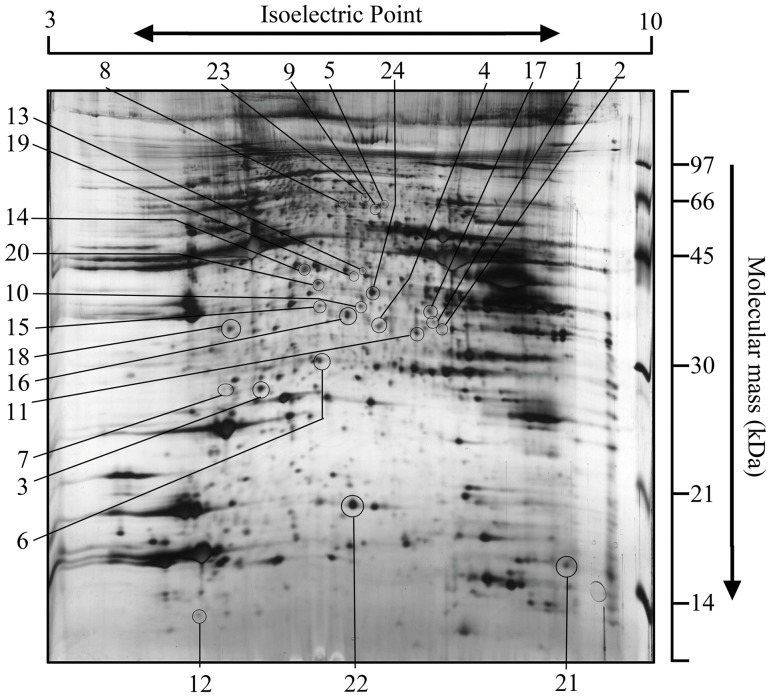
Quadriceps femoris protein profiling by 2-DE. A typical 2-D-pattern gel image of 100-µg protein extract separated in a pH 3–10 IPG strip in the first dimension and 13% polyacrylamide gel in the second dimension. One 2-D gel was performed each sample, 6 samples per group. Twenty-five differentially expressed (p<0.05) spots were labeled with spot number as they appear in the MS list (see Table 2).

Protein separation was performed by 2-DE. Fig. 4 shows a representative image of skeletal muscle proteins. Protein identified by MALDI-TOF-MS or LC-MS/MS are listed in Table 2. Image analysis of gels revealed the presence of protein spots, visualized by silver staining. In the present study, we used an NE (normal chow, exercise) group for a control (See Table S1) to characterize the exercise effects on mice with normal diet as opposed to the exercise effects on mice with high-fat diet. Fifteen protein spots were significantly altered between NC and HC, including one spot that disappeared exclusively in HC. Twenty-three protein spots were significantly changed between HC and HE, including one spot that disappeared and one spot that appeared exclusively in HE group. Fourteen spots were altered in opposite by high-fat diet and aerobic exercise. Proteins involved in the biological processes of transport, protein synthesis and degradation, muscle contractile, carbohydrate metabolism, oxidative stress response, and others underwent Gene Ontology analysis. In the present study, some proteins were present in multiple spots, such as spot 8, 9, 10, 11 and 13. Two spots (spot 8 and 9) were identified as Trim 72, and three spots (spot 10, 11, and 13) were identified as MHC IIb, which are perhaps due to isoforms of the same proteins.

**Table 2:** List of identified protein by LC-MS/MS or MALDI-TOF/MS.

Spot No.	Protein Name	Description	GI Number	Score	Sequence coverage	Matched peptides	MW^a^	Pl^b^	HC vs. NC		HE vs. HC	
									Fold Change	P value	Fold Change	P value
**Transport**												
1*	Vdac2	Voltage-Dependent Anion-Selective Channel Protein 2	6755965	400	33%	10	31713	7.44	3.96	0.007	−1.72	0.016
2*	Vdac1	Voltage-Dependent Anion-Selective Channel Protein 1	6755963	146	16%	5	30737	8.62	3.56	0.042	−2.38	0.009
3	Apoa1bp	Apolipoprotein A-I Precursor	109571	249	48%	19	30358	5.52	−5.56	0.029	2.62	0.021
4	Fabp4	Chain A, C1Gv32Df57H Mutant Of Murine AdipocyteLipid Binding Protein At pH 4.5	157829776	92	45%	8	14469	8.01	1.52	<0.001	−2	0.013
**Protein synthesis and degradation**												
5*	Cct2	CCT (Chaperonin Containing Tcp-1) Beta Subunit	468546	379	22%	8	57411	5.97	NS		−2	0.037
6	Hspb1	Heat Shock Protein Beta-1	158937312	160	58%	12	23000	6.12	−4.95	0.045	1.73	0.025
7	Psma1	Proteasome Subunit Alpha Type-1	33563282	89	36%	8	29528	6	NS		1.67	0.014
**Muscle Contractile**												
8*	Trim72	Trim72 Protein	121247302	139	8%	3	52783	6.01	NS		−3.7	0.033
9*	Trim72	Trim72 Protein	121247302	78	24%	8	52783	6.01	NS		−1.78	0.015
10*	Myh4	Myosin Heavy Chain IIb	9581821	159	16%	7	60994	5.38	−2.63	0.037	2.69	0.002
11*	Myh4	Myosin Heavy Chain IIb	9581821	440	21%	11	60994	5.38	−8.33	0.041	8.27	0.049
12*	Mylpf	Myosin Regulatory Light Chain 2, SkeletalMuscle Isoform	7949078	104	11%	2	18943	4.82	NS		1.8	0.004
13*	Myh4	Myosin Heavy Chain IIb	9581821	312	16%	8	60994	5.38	−3.33	0.026	10.08	0.048
**Carbohydrate Metabolism**												
14*	Eno3	Beta-Enolase Isoform 1	6679651	270	36%	12	46995	6.73	−5.56	0.042	2.26	0.027
15*	Eno1	Enolase 1, Alpha Non-Neuron	123244133	46	43%	2	7353	6.56	−5.26	0.046	7.51	0.039
16*	Hibadh	3-Hydroxyisobutyrate Dehydrogenase,Mitochondrial Precursor	21704140	62	14%	3	35417	8.37	−5.88	0.005	4.21	0.010
17*	Gapdh	Glycerol-3-Phosphate Dehydrogenase	387177	126	29%	8	37560	6.75	−2.7	<0.001	NS	
18*	Coq9	Ubiquinone Biosynthesis Protein COQ9,Mitochondrial Precursor	33859690	177	29%	6	35061	5.6	−1.96	0.034	1.61	0.002
19	Ldh	L-Lactate Dehydrogenase B Chain	6678674	101	23%	9	36549	5.7	2.16	0.006	−2.78	0.031
20	Mdh1	Malate Dehydrogenase, Cytoplasmic	254540027	155	39%	13	36488	6.16	NS		−2.94	0.006
21	Uqcrfs1	Cytochrome B-C1 Complex Subunit Rieske, Mitochondrial	13385168	96	17%	8	29349	8.91	NS		−2.27	0.015
**Oxidative stress response**												
22*	Sod1	Cu/Zn Superoxide Dismutase	226471	128	16%	2	15752	6.03	Disappear	<0.001	Appear	<0.001
**Others**												
23	Fgb	Fibrinogen Beta Chain Precursor	33859809	232	45%	22	54718	6.68	NS		−2.63	0.021
24		Predicted: Hypothetical Protein	83011571	66	49%	6	9456	9.69	NS		−2.56	0.024

* Spot identified by LC-MS/MS.

a Theoretical molecular mass.

b Theoretical pI.

NS: No significant difference between NC and HC, or HC and HE group.

### Immunoblot Analysis

**Figure 5:**
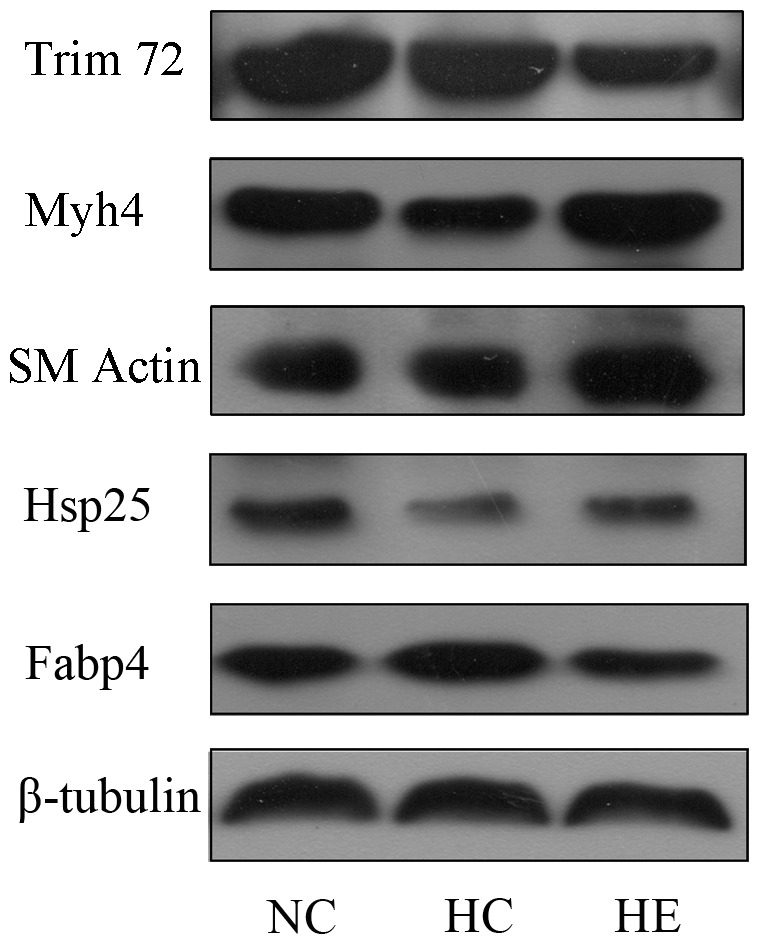
Selected proteins from 2-DE were confirmed by immunoblot analysis. Expression of Trim72, Myh4, Skeletal Muscle Actin (SM Actin), Hsp25 and Fabp4 were assessed by western blot analysis of skeletal muscle proteins from NC, HC, and HE mice; β-tubulin was used as an internal control for loading.

**Figure 6:**
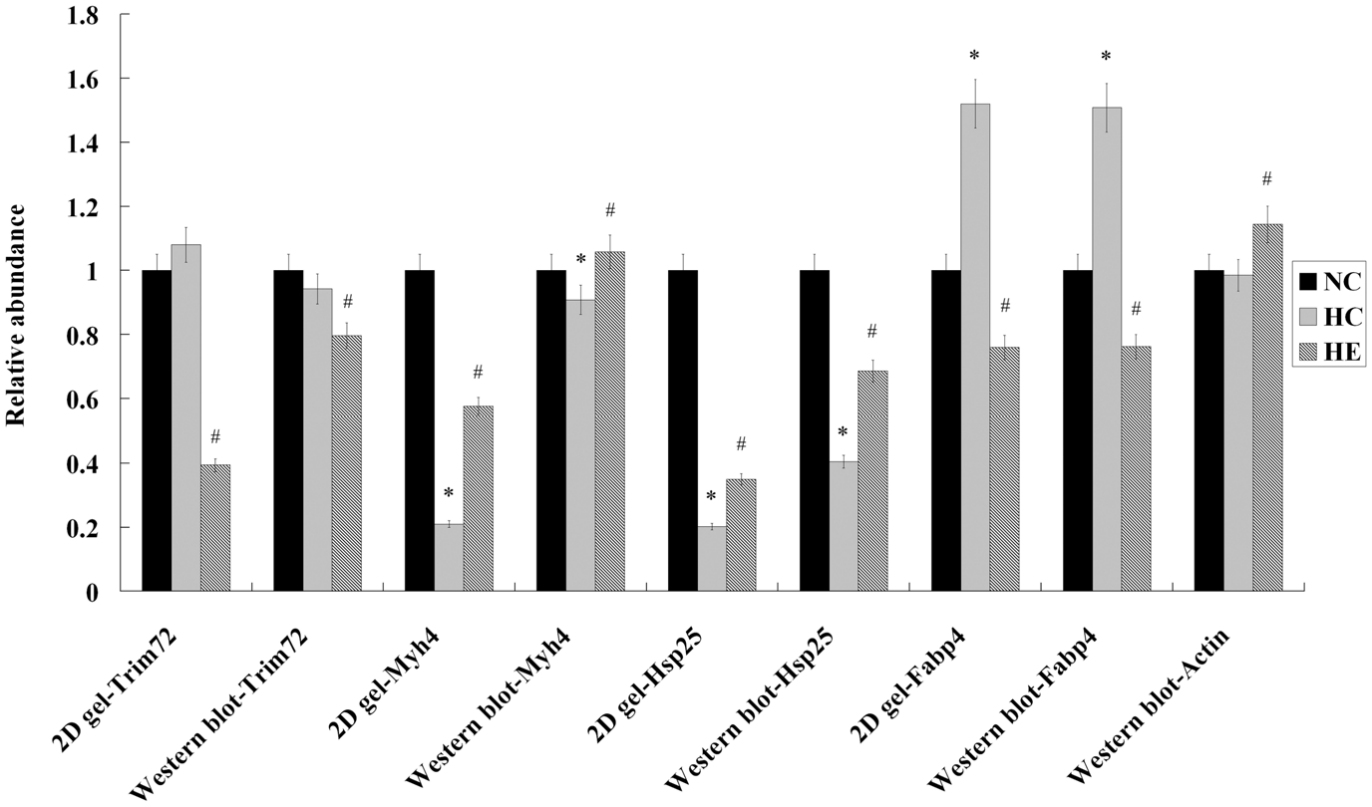
Relative abundance of the 2D gels and immunoblots. Results are means ± SEM (n = 6, each group). *: p<0.05 HFD control (HC) vs. normal chow (NC); #: p<0.05 HFD exercise (HE) vs. HFD control (HC).

Although our proteomic data indicated differential protein expression among all the groups, we could not exclude the possibility of false-positive findings in the proteomic analysis. To address this issue, some proteins (Fig. 5), including Fabp4, Hsp25, Myh4 and Trim72 were further confirmed by immunoblot analysis. As shown in Fig. 6, the expression levels of all tested proteins were basically consistent with those of the proteomic study (Also shown as Figure S1). To determine whether the skeletal muscle mass was enhanced by exercise training, we also detected skeletal muscle actin by western blot (Figs. 5 and 6). As expected, skeletal muscle actin was significantly increased by 6-week aerobic exercise, which is consistent to the change of Myh4 between HC and HE group.

## Discussion

Impaired insulin action on the whole-body glucose uptake is a hallmark feature of IR. Aerobic exercise has been linked to improved glucose homeostasis and enhanced insulin sensitivity in humans and rodents [21], [22]. In this study, we showed that moderate intensity exercise training decreased body weight and improved the abnormal lipid profile induced by high-fat diet. We also found that fasting serum insulin and impaired glucose tolerance were attenuated by 6 weeks of aerobic exercise. These changes are consistent with the previous studies [23], [24] and revealed that aerobic exercise plays a key role in body weight control, glucose homeostasis, and the blood lipid profile. Citrate synthase has been routinely used to assess oxidative capacity and mitochondrial density. To determine the effect of prolonged exercise training on muscle oxidative capacity, we detected citrate synthase activity of skeletal muscle in each group. Our result shows that the exercise protocol of 6-week treadmill training enhanced citrate synthase activity, revealing increased oxidative capacity in HE group.

Previous studies of proteomics regarding the response of skeletal muscle to exercise training have shown a lot of differentially expressed proteins [16], [17]. The present study results extend this to show a global protein expression profile of the skeletal muscle response of IR mice to aerobic exercise. Our previous microarray analysis screened a large number of differential expressed genes involved in metabolism, defense, and inflammation and genes of unknown function [12]. The current study also showed changes of the proteins involved in metabolism including energy metabolism, mitochondrial functions. In addition, the proteins that involved in muscle contractile, oxidative stress response and protein folding and degradation were changed as well.

Results from the present study show abundant changes of several proteins involved in function of chaperonin proteins, namely Hsp25 and CCT (chaperonin containing TCP-1) Beta Subunit (Cct2). Hsp25 is a member of the small heat shock proteins family, which has been shown to protect different types of cells against oxidative stress [25]. Overexpression of Hsp25 protected L929 cells against TNFα-induced ROS production, protein oxidation, and cell death [26]. In the present study, the abundance of Hsp25 protein in IR mice was significantly increased by 6-week aerobic exercise. Interestingly, Cu/Zn superoxide dismutase (Sod1), regarded as an antioxidant that changed in coordination with Hsp25 abundance, was also increased by exercise training in the present study, suggesting that the exercise-induced increase of Hsp25 may play a key role in improving IR by preventing oxidative stress.

Another chaperonin protein observed in the present study is Cct2, which is one of eight different subunits (CCT α, β, γ, δ, ε, ζ, η, and θ, the equivalent of CCT1, 2, 3, 4, 5, 6, 7, and 8). Available data suggests that Cct2 mediates protein folding involved in cytoskeletal formation and contractile activity [27], [28]. Cct2 has recently been identified as a novel physiological substrate for p70 ribosomal S6 kinase 1 (S6K1), which is the downstream molecular of mammalian target of rapamycin (mTOR) [29]. Insulin activates the phosphoinositide 3-kinase (PI3K)-mTOR pathway and utilizes S6K1 to regulate CCT phosphorylation [30]. Our results show that aerobic exercise decreases the expression of Cct2 in skeletal muscle of IR mice. Although the exact molecular mechanism of Cct2 regulation remains unclear, our findings demonstrate a link between Cct2 and aerobic exercise in the development of IR. We hypothesize that aerobic exercise might increase the protein folding activity of Cct2 through the mTOR/S6K1 pathway, thereby reducing potential unfolded protein stress response and improving IR.

Another novel change observed in the present study is that 6-week aerobic exercise significantly reversed the high expression level of Fabp4 in the skeletal muscle of IR mice. Fabp4 protein is abundantly expressed in a tissue-specific manner, and it was originally identified as an adipocyte- and macrophage-specific protein [31] that is capable of binding a variety of hydrophobic ligands, such as long-chain fatty acids, eicosanoids, leukotrienes, and prostaglandins [32], [33]. However, recent studies suggest it may be more widely expressed, such as in the endothelial cells of heart and kidney, plasma, skeletal muscle and other types of cells [34]–[36]. Numerous investigations reveal that Fabp4 plays an important role in maintaining glucose and lipid homeostasis [37], [38]. Deficiency of Fabp4 has been correlated with a lack of insulin sensitivity in obese mice [39]. Fabp4 is especially protective against diet-induced IR [40]. Fabp4 plasma concentration was also reported to be increased with the early presence of metabolic syndrome in humans [41]. Exercise training with weight loss induced a significant decrease in plasma Fabp4 levels in obese women [42]. However, most studies regarding the association between Fabp4 and obesity/IR have focused on the serum or adipocyte rather than skeletal muscle Fabp4; the exact mechanisms by which Fabp4 regulates different biological functions in skeletal muscle are not well understood. Our results showed that aerobic exercise decreased the abundance of Fabp4 protein in the insulin resistant skeletal muscle. Improved plasma lipid profile with the reduction in plasma TG and TC levels were also observed. Therefore, down-expression of Fabp4 in skeletal muscle is believed to play an important role in fatty acid metabolism and the avoidance of intramyocyte lipotoxicity, which is a main cause of IR in skeletal muscle.

The present findings also show changes in the abundance of myofibrillar contraction, namely Trim72 and myosin heavy chain IIb. Trim72 is specifically expressed in the plasma membrane of skeletal muscle and plays a critical role in membrane repair response to acute muscle injury [43]. Although the role of Trim72 in muscle adaptation to aerobic exercise is not fully understood, Trim72 negatively regulates myogenesis by fortifying IgF-I-mediated IRS-1 activation in C2C12 cell during muscle differentiation [44]. Our results show that Trim72 protein expression significantly decreased after 6-week aerobic exercise, while myosin heavy chain IIb and actin expression significantly increased in skeletal muscle, indicating that exercise training may enhance skeletal muscle mass via regulation of the expression of Trim72. Thus, Trim72 could be a useful therapeutic target in obesity, IR, and T2DM because insulin sensitivity and glucose uptake is highly increased in the enhanced skeletal muscle.

### Conclusions

Our results demonstrate a wide array of changes in protein abundance in exercise-trained skeletal muscle, which provide the basis for new hypotheses regarding the mechanism of IR improved by aerobic exercise. These potential themes include alterations in abundance of proteins involved in molecular chaperones, antioxidative stress response, lipid binding, myofibrillar contraction, mitochondrial functions. These underlying mechanisms need to be tested in future study.

## Supporting Information

Figure S1 **Selected proteins from 2-DE were confirmed by immunoblot analysis.** More data regarding the protein expression levels of Trim72, Myh4, Skeletal Muscle Actin (SM Actin), Hsp25 and Fabp4 analyzed by western blot were shown; β-tubulin was used as an internal control for loading.
(TIF)

Table S1 **List of identified protein by LC-MS/MS or MALDI-TOF/MS (NC and NE).** An NE (normal chow, exercise) group was used for a control to characterize the exercise effects on mice with normal diet as opposed to the exercise effects on mice with high-fat diet. The changes of spots t between NC and NE were shown.
(DOC)
