# Supplementary figures and images for: Proteomic Analysis of Skeletal Muscle in Insulin-Resistant Mice: Response to 6-Week Aerobic Exercise
Source: PLoS One. 2013 Jan 9;8(1):e53887. doi: 10.1371/journal.pone.0053887 (PMC3541238; doi:10.1371/journal.pone.0053887)

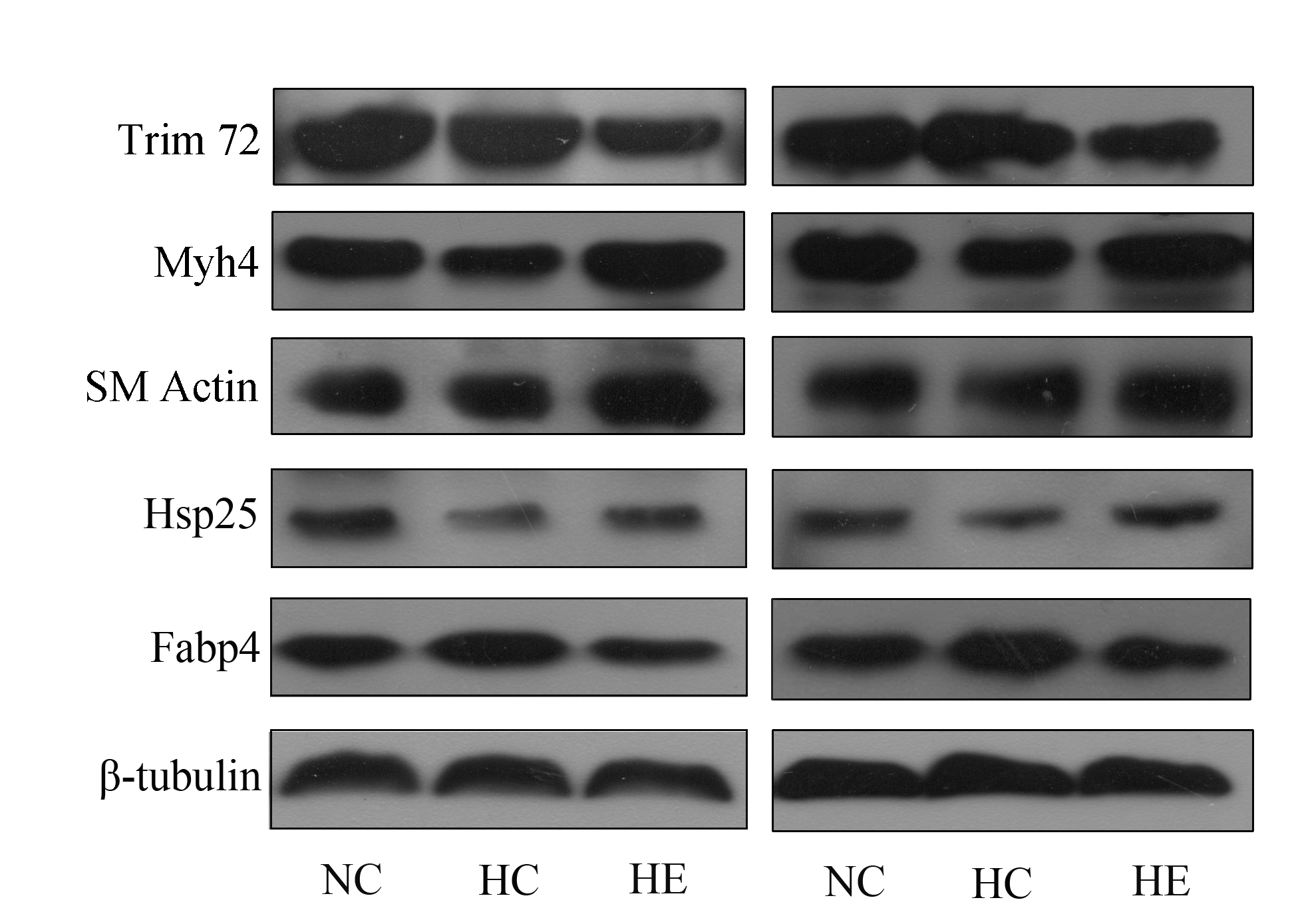

Supplement: Figure S1 — Selected proteins from 2-DE were confirmed by immunoblot analysis. More data regarding the protein expression levels of Trim72, Myh4, Skeletal Muscle Actin (SM Actin), Hsp25 and Fabp4 analyzed by western blot were shown; β-tubulin was used as an internal control for loading. (TIF) [file pone.0053887.s001.tif]
