# Supplementary material for: Proteomic Analysis of Skeletal Muscle in Insulin-Resistant Mice: Response to 6-Week Aerobic Exercise
Source: PLoS One. 2013 Jan 9;8(1):e53887. doi: 10.1371/journal.pone.0053887 (PMC3541238; doi:10.1371/journal.pone.0053887)
Supplement: Table S1 — List of identified protein by LC-MS/MS or MALDI-TOF/MS (NC and NE). An NE (normal chow, exercise) group was used for a control to characterize the exercise effects on mice with normal diet as opposed to the exercise effects on mice with high-fat diet. The changes of spots t between NC and NE were shown. (DOC) [file pone.0053887.s002.doc]

**Table S1 List of identified protein by LC-MS/MS or** **MALDI-TOF/MS (NE vs. NC)**

|  | Protein Name | Description | GI Number | Score | Sequence coverage | Matched peptides | MW ^a^ | Pl ^b^ | NE vs. NC | |
| --- | --- | --- | --- | --- | --- | --- | --- | --- | --- | --- |
|  |  |  |  |  |  |  |  |  | Fold Change | P value |
| **Transport** | | | | | | | | | | |
| 1 ^*^ | Vdac2 | Voltage-Dependent Anion-Selective Channel Protein 2 | 6755965 | 400 | 33% | 10 | 31713 | 7.44 | -2.34 | 0.015 |
| 2 ^*^ | Vdac1 | Voltage-Dependent Anion-Selective Channel Protein 1 | 6755963 | 146 | 16% | 5 | 30737 | 8.62 | -1.93 | 0.019 |
| 3 | Apoa1bp | Apolipoprotein A-I Precursor | 109571 | 249 | 48% | 19 | 30358 | 5.52 | 3.01 | 0.043 |
| 4 | Fabp4 | Chain A, C1Gv32Df57H Mutant Of Murine Adipocyte Lipid Binding Protein At pH 4.5 | 157829776 | 92 | 45% | 8 | 14469 | 8.01 | -2.56 | 0.048 |
| **Protein synthesis and degradation** | | | | | | | | | | |
| 5 ^*^ | Cct2 | CCT (Chaperonin Containing Tcp-1) Beta Subunit | 468546 | 379 | 22% | 8 | 57411 | 5.97 | -3.98 | 0.034 |
| 6 | Hspb1 | Heat Shock Protein Beta-1 | 158937312 | 160 | 58% | 12 | 23000 | 6.12 | 2.14 | 0.029 |
| 7 | Psma1 | Proteasome Subunit Alpha Type-1 | 33563282 | 89 | 36% | 8 | 29528 | 6 | 3.58 | 0.018 |
| **Muscle Contractile** | | | | | | | | | | |
| 8 ^*^ | Trim72 | Trim72 Protein | 121247302 | 139 | 8% | 3 | 52783 | 6.01 | -2.1 | 0.009 |
| 9 ^*^ | Trim72 | Trim72 Protein | 121247302 | 78 | 24% | 8 | 52783 | 6.01 | -1.93 | 0.020 |
| 10 ^*^ | Myh4 | Myosin Heavy Chain IIb | 9581821 | 159 | 16% | 7 | 60994 | 5.38 | 1.57 | 0.039 |
| 11 ^*^ | Myh4 | Myosin Heavy Chain IIb | 9581821 | 440 | 21% | 11 | 60994 | 5.38 | 3.94 | 0.041 |
| 12 ^*^ | Mylpf | Myosin Regulatory Light Chain 2, Skeletal Muscle Isoform | 7949078 | 104 | 11% | 2 | 18943 | 4.82 | 2.1 | 0.014 |
| 13 ^*^ | Myh4 | Myosin Heavy Chain IIb | 9581821 | 312 | 16% | 8 | 60994 | 5.38 | 2.88 | 0.039 |
| **Carbohydrate Metabolism** | | | | | | | | | | |
| 14 ^*^ | Eno3 | Beta-Enolase Isoform 1 | 6679651 | 270 | 36% | 12 | 46995 | 6.73 | 3.34 | 0.023 |
| 15 ^*^ | Eno1 | Enolase 1, Alpha Non-Neuron | 123244133 | 46 | 43% | 2 | 7353 | 6.56 | 4.08 | 0.038 |
| 16 ^*^ | Hibadh | 3-Hydroxyisobutyrate Dehydrogenase, Mitochondrial Precursor | 21704140 | 62 | 14% | 3 | 35417 | 8.37 | 2.91 | 0.028 |
| 17 ^*^ | Gapdh | Glycerol-3-Phosphate Dehydrogenase | 387177 | 126 | 29% | 8 | 37560 | 6.75 | 1.82 | 0.003 |
| 18 ^*^ | Coq9 | Ubiquinone Biosynthesis Protein COQ9, Mitochondrial Precursor | 33859690 | 177 | 29% | 6 | 35061 | 5.6 | 2.26 | 0.009 |
| 19 | Ldh | L-Lactate Dehydrogenase B Chain | 6678674 | 101 | 23% | 9 | 36549 | 5.7 | -1.69 | 0.037 |
| 20 | Mdh1 | Malate Dehydrogenase, Cytoplasmic | 254540027 | 155 | 39% | 13 | 36488 | 6.16 | -3.08 | 0.013 |
| 21 | Uqcrfs1 | Cytochrome B-C1 Complex Subunit Rieske, Mitochondrial | 13385168 | 96 | 17% | 8 | 29349 | 8.91 | -2.19 | 0.032 |
| **Oxidative stress response** | | | | | | | | | | |
| 22 ^*^ | Sod1 | Cu/Zn Superoxide Dismutase | 226471 | 128 | 16% | 2 | 15752 | 6.03 | 1.97 | 0.003 |
| **Others** | | | | | | | | | | |
| 23 | Fgb | Fibrinogen Beta Chain Precursor | 33859809 | 232 | 45% | 22 | 54718 | 6.68 | -1.74 | 0.008 |
| 24 |  | Predicted: Hypothetical Protein | 83011571 | 66 | 49% | 6 | 9456 | 9.69 | -3.46 | 0.017 |

^*^ Spot identified by LC-MS/MS.

^a^ Theoretical molecular mass.

^b^ Theoretical pI.
